# The Neural Responses to Social Cooperation in Gain and Loss Context

**DOI:** 10.1371/journal.pone.0160503

**Published:** 2016-08-05

**Authors:** Peng Sun, Li Zheng, Lin Li, Xiuyan Guo, Weidong Zhang, Yijie Zheng

**Affiliations:** 1 School of Psychology and Cognitive Science, East China Normal University, Shanghai, China; 2 Key Laboratory of Brain Functional Genomics, Ministry of Education, Shanghai Key Laboratory of Brain Functional Genomics, School of Psychology and Cognitive Science, East China Normal University, Shanghai, China; 3 Shanghai Key Laboratory of Magnetic Resonance, School of Psychology and Cognitive Science, East China Normal University, Shanghai, China; 4 Key Laboratory of Brain Functional Genomics, Ministry of Education, Shanghai Key Laboratory of Brain Functional Genomics, East China Normal University, Shanghai, China; 5 Shanghai Key Laboratory of Magnetic Resonance, Department of Physics, East China Normal University, Shanghai, China; Radboud University Nijmegen, NETHERLANDS

## Abstract

Cooperation is pervasive and constitutes the core behavioral principle of human social life. Previous studies have revealed that mutual cooperation was reliably correlated with two reward-related brain regions, the ventral striatum and the orbitofrontal cortex. Using functional magnetic resonance imaging (fMRI), this study sought to investigate how the loss and gain contexts modulated the neural responses to mutual cooperation. Twenty-five female participants were scanned when they played a series of one-shot prisoner’s dilemma games in the loss and gain contexts. Specifically, participants and partners independently chose to either cooperate with each other or not, and each was awarded or deprived of (in the gain context or the loss context, respectively) a sum of money which depended upon the interaction of their choices. Behavioral results indicated that participants cooperated in nearly half of the experiment trials and reported higher level of positive emotions for mutual cooperation in both contexts, but they cooperated more in the gain than in the loss context. At the neural level, stronger activities in the orbitofrontal cortex were observed for mutual cooperation compared with the other three outcomes in both contexts, while stronger activation in ventral striatum associated with mutual cooperation was observed in the gain context only. Together, our data indicated that, even in the one-shot interaction under loss context, participants still exhibited preference for cooperation and the rewarding experience from a mutually cooperative social interaction activated the ventral striatum and the orbitofrontal cortex, but the loss context weakened the association between the ventral striatum activation and mutual cooperation.

## Introduction

Traditional economic theories generally assume that people are absolutely self-interested. However, hundreds of recent experimental evidences have indicated that, in social interactions, people often showed concerns for interests of others and exhibited strong preferences for fairness and reciprocity, eventually leading to regular deviations from purely self-interested behaviors [1–3]. A good example is the pervasive cooperation among nonrelatives, which is an important base of human society, especially when the social division of labor is striding towards intensification and specialization.

Cooperative behavior among nonrelatives has been widely explored in behavioral and neuroimaging studies using Prisoners’ Dilemma (PD) game. In this game, two players simultaneously decide whether or not to cooperate with each other and receive a payoff that depends upon the interaction of their respective choices. Mutual cooperation leads to a modest payoff to both players, while a lesser amount of payoff to both players would occur in case of mutual defection. Importantly, a player gets largest payoff when he defects and the partner cooperates, and the worst outcome is got when the player cooperates while the partner defects. According to traditional economic theories, for maximizing personal benefits, the player should always defect. However, abundant studies have found that players cooperated more than expected, with mutual cooperation occurring about 50% [4]. Even when the interaction was anonymous and one-shot, which mean there were no monetary return and gain for reputation in the future, a considerable percentage of people still tend to cooperate [5]. How did people resist the temptation of monetary rewards and choose to cooperate with nonrelatives in the PD game? To answer this question, a large number of functional magnetic resonance imaging (fMRI) studies have been conducted using the PD game or related trust games. It was revealed that mutual cooperation was reliably correlated with two reward-related brain regions, the ventral striatum and the orbitofrontal cortex [6–9]. Moreover, the degree of future cooperation could be predicted by the activation in the ventral striatum [6, 10]. Therefore, based on these findings, some researchers speculated that cooperation, as a type of social reward represented in the ventral striatum and the orbitofrontal cortex, is inherently rewarding and has higher subjective value than monetary rewards gained from defection [2].

However, previous neuroimaging studies were usually conducted with repeated PD game, which leaving open the possibility that activations in reward regions were the result of that participants anticipated getting more money from the cooperation partnership in the future. In addition, most of previous studies using the PD game were conducted in gain context, i.e. players were awarded a sum of money depended upon the interaction of their choices. But, in real world, there also exist many situations in which people had to share a certain amount of loss, such as the failure of co-investments. It remains unclear whether people still endow higher subjective value to mutual cooperation than monetary rewards gained from defection when the conflict between personal and others’ interests concentrates on negative outcomes.

To shed light on these questions, in the present fMRI study, we adopted a variant of the one-shot PD game in which loss context was employed. Specifically, participants and partners independently chose to either cooperate with each other or not, and each was awarded or deprived of (in the gain context or the loss context, respectively) a sum of money that depended upon the interaction of their choices. Following a conventional approach to study context effect on decision making, participants in the current study were initially endowed with 100 RMB as basic payment and the losses incurred were subtracted from this initial endowment. In the version of the game we used here, it was played a single time with each other. Firstly, we aimed to explored whether mutual cooperation was still associated with activations of brain regions involved in reward processing in the one-shot and anonymous interaction, in which there were no long-term benefits being expected from a cooperation partnership. Secondly, we sought to investigate the mechanism underlying the modulation of responses to cooperation in the PD game by context (loss vs. gain). Based on previous findings, we hypothesized that, even in the one-shot and anonymous interaction, activations in brain region implicated reward processing (i.e. ventral striatum and orbitofrontal cortex) still existed for mutual cooperation, but the activation strength might be different between the gain and the loss context.

## Materials and Methods

### Participants

Twenty-five right-handed female volunteers from the university community with normal or corrected-to-normal vision participated in this experiment. For imaging data analyses, three participants were excluded from further statistical analysis because of lack of cooperated trials or defected trials in at least one condition. One of them did not cooperate at all. One of them did not give cooperation responses in the gain context and the last one did not give defection responses in the gain context. In addition, the other four participants had to be excluded due to excessive head movements, finally leaving 18 subjects for imaging data analyses [mean age = 21.04 ± 1.59 (SD) years]. None of the participants reported a significant abnormal neurological history. All the participants gave written informed consent before scanning and were paid according to outcomes from a random selection of 20% trials plus a 100 RMB bonus. This study was approved by the Ethical Committee of East China Normal University.

### Materials

Eighty female face pictures with neutral expression were selected from the Chinese Facial Affective Picture System[11] with consent for publication, and randomly allocated to 2 (context: loss vs. gain) × 2 (type of partner: cooperated vs. defected) conditions. The arousal and attractiveness of pictures were counterbalanced across different conditions.

### Procedure

Before scanning, participants were told the rules of the game and that they would play with 80 different strange female partners who are students from another university in Shanghai. They were also informed that, for practical reasons, not all 80 game partners could actually be present in the fMRI laboratory, but that partners’ choices had been collected before the experiment. In addition, participants were told that both she and the partner in each trial would be paid according to the interaction of their choices. They would be given a basic payment for their participation (100 RMB) plus or minus the amount of money obtained or lost from a random selection of 20% trials in the game. In reality, each subject was the only participant in the experiment, and putative partners’ choices were determined in advance, so that half of partners cooperated and the other half defected in each context.

**Fig 1:**
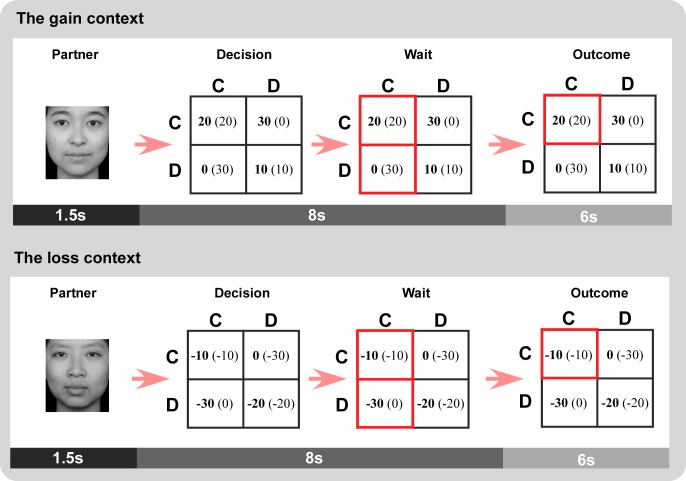
Experimental procedure. Participants were scanned while playing the game for 80 trials, 40 in each context. Participant’s choices (C or D) are listed atop columns and partner’s choices (C or D) are listed aside rows. Money amounts in bold are for participant. Amounts in parentheses are for partner.

The participants then completed 80 trials in the scanner with 40 trials in each context (Fig 1). Each trial began with a 1.5s presentation of the partner's face. During the next 8s epoch, participants chose to cooperate or defect by pressing one of two buttons on a button box, and their choices were showed in red. After that, the trial outcome was displayed for 6s. All the trials were presented in a random order. Each trial was jittered with inter-stimulus intervals from 2 to 5 s, during which a black fixation cross was presented. Two additional jittered blanks (750∼2000ms; 1500∼2500ms) were set between the presentation of the partner’s face and the decision phase and, between the wait phase and the outcome phase.

After scanning, participants were asked to rate their emotional reactions to four PD game outcomes (CC: player and partner both cooperated; CD: player cooperated and partner defected; DC: player defected and partner cooperated; DD: player and partner both defected) in each context. Nine point Likert scales were used to rate the following emotions/feelings: happiness, trust, anger, disappointment, shame, and guilt.

### fMRI Image Acquisition and Analysis

Scanning was carried out on a 3T Siemens scanner at the Functional MRI Lab (East China Normal University, Shanghai). For functional images, 36 slices were acquired using a gradient-echo echo-planar imaging (EPI) sequence (TR = 2200 ms, TE = 30 ms, FOV = 220 mm, matrix size = 64 × 64, slice thickness = 3 mm, gap = 0.3 mm). Before the functional run, a high-resolution structural image was acquired using a T1-weighted, multiplanar reconstruction (MPR) sequence (TR = 1900 ms, TE = 3.42 ms, 192 slices, slice thickness = 1 mm, FOV = 256 mm, matrix size = 256 × 256).

Data pre-processing and statistical analyses were performed with Statistical Parametric Mapping (SPM8, Wellcome Department of Cognitive Neurology, London). The functional images were corrected for the delay in slice acquisition and were realigned to the first image to correct for interscan head movements. The individual T1-weighted, 3D structural image was co-registered to the mean EPI image generated after realignment. The co-registered structural image was then segmented into gray matter (GM), white matter (WM) and cerebrospinal fluid (CSF) using a unified segmentation algorithm[12].The functional images after slice timing and realignment procedures were spatially normalized to the Montreal Neurological Institute (MNI) space (resampled at 2 × 2 × 2 mm^3^) using the normalization parameters estimated during unified segmentation and then spatially smoothed with a Gaussian kernel of 8 mm full-width half-maximum (FWHM).

A general linear model (GLM) was defined for each participant that examined the neural response to the period in which outcome was revealed. More specially, at the first level, eight types of events were defined according to context (Loss vs. Gain) and the PD game outcome (CC, CD, DC, and DD). They were convolved using a canonical hemodynamic response function. All the encoding trials were modeled from the onset time of the outcomes (with zero duration). Additional regressors of no interest were created for partner presentation, decision and wait. Six regressors modeling movement-related variance and one modeling the overall mean were also employed in the design matrix. A general linear model analysis created eight contrast images for each participant summarizing differences of interest. The eight first level contrast images from each participant were then analyzed at the second level employing a random-effects model (flexible factorial design in SPM8).

We performed region of interest (ROI) analyses on the ventral striatum and the orbitofrontal cortex which involved in reward processing. These two ROIs were defined as 10 mm-radius sphere centered on the activation peak for the contrast between mutual cooperation and the average of the other three PD game outcomes reported in Rilling et al.[6, 7, 13], after converting from Talairach to MNI coordinates. Parameter estimates were extracted from each ROI for each of the eight experimental conditions from each participant using MarsBaR and then entered into ANOVA.

This ROI analysis was supplemented with a whole brain analysis in which brain activities related to mutual cooperation were defined by contrasting the BOLD response to the CC outcome with the average response of the other three outcomes combined[6, 7, 13]. Brain activations corresponding to gain relative to loss trials were identified by the (Gain—Loss) and reverse contrasts. Then, the context × cooperation interactions defined by (CC—Average of the other three outcomes)_gain_—(CC—Average of the other three outcomes)_loss_ were computed to explore how context affect cooperation. Areas of activations were identified as significant only if they passed the threshold of *p* < 0.05 family-wise error (FWE) corrected for multiple comparisons at the cluster-level with an underlying voxel-level of *p* < 0.001 (uncorrected).

## Results

### Behavioral results

First of all, we examined participants’ strategy during the task to make sure that their responses in the PD game were not just randomly decided. It turned out that participants did not adopt a Win-Stay/Lose-Shift strategy or Tit-for-Tat-like strategies. Instead, our data revealed participants’ decision were influenced by their own choices in the preceding trial. Specially, Paired-sample t test showed that participants cooperated more after defecting (*M* = 0.45) than cooperating (*M* = 0.35; *t*(24) = -3.47, *p* = 0.002) in the preceding trial.

Paired-sample t test and χ^2^ test revealed a greater cooperation rates when participants played the PD game in the gain context (*M* = 0.46, *SD* = 0.27) than in the loss context (*M* = 0.34, *SD* = 0.17) (*t*(24) = 3.27, *p =* 0.003; χ^2^_1_ = 32.06, P < 0.001).

**Fig 2:**
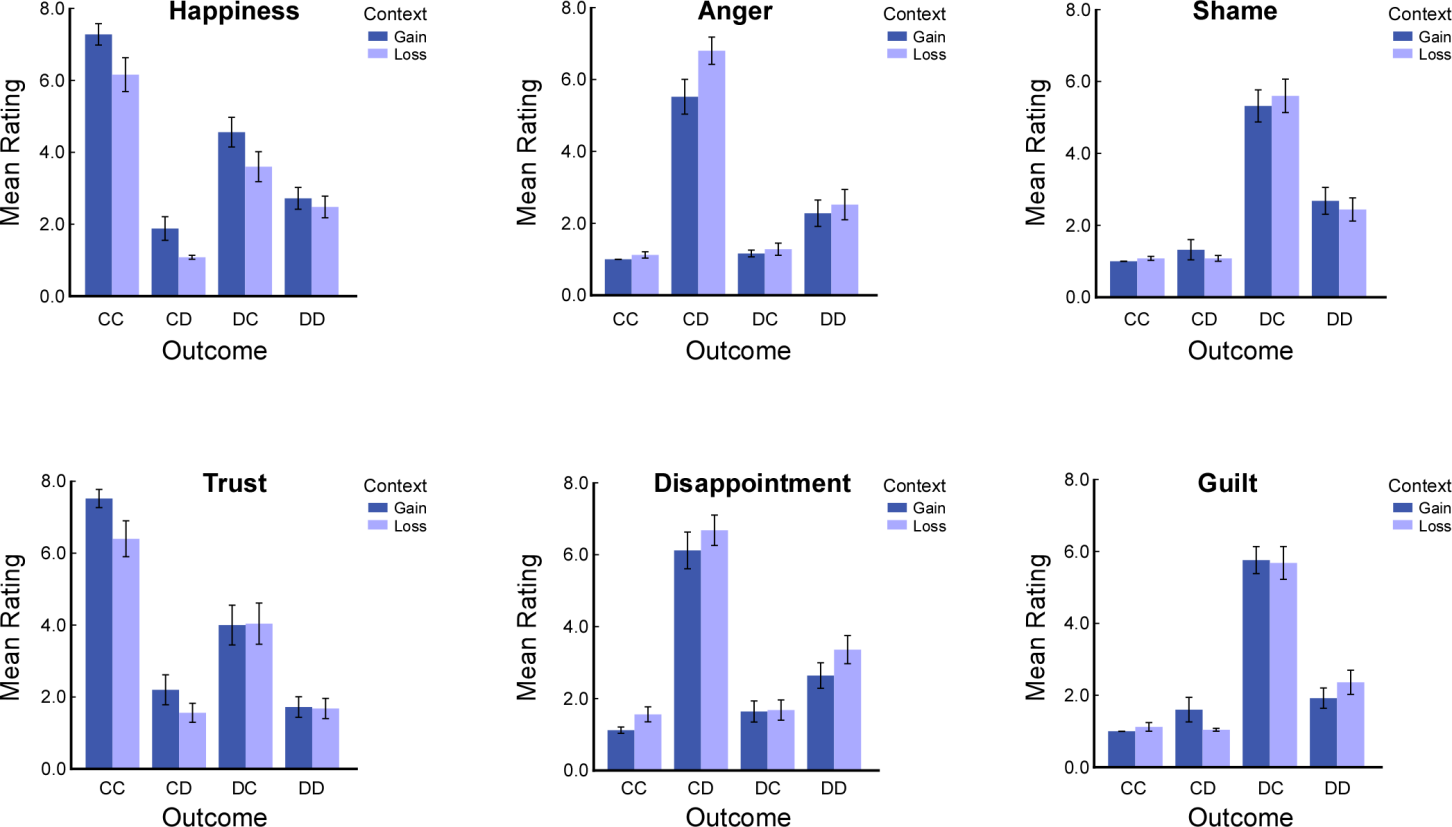
Emotion ratings for each outcome of the Prisoner’s Dilemma game in the gain and the loss context. C = cooperate; D = defect.

A 2 (context: loss vs. gain) × 4 (outcome: CC, CD, DC, and DD) repeated measures ANOVA was carried out for each emotion rating (Fig 2). Specially, for ratings of happiness, there were significant main effects of context (*F*(1,24) = 9.79, *p =* 0.005) and outcome (*F*(3,72) = 67.23, *p <* 0.001). Post hoc analyses showed that the level of happiness was higher for CC outcome than the other three outcomes (*ps <* 0.001) and higher in the gain than the loss context (*p =* 0.005). For ratings of trust, there were significant main effects of outcome (*F*(3,72) = 54.89, *p <* 0.001), post hoc analyses showed that the level of trust was higher for CC outcome than the other three outcomes (*ps <* 0.001). For ratings of anger and disappointment, there were significant main effects of context(anger: *F*(1,24) = 5.87, *p =* 0.023; disappointment: *F*(1,24) = 5.07, *p =* 0.034) and main effects of outcome (anger: *F*(3,72) = 74.14, *p <* 0.001; disappointment: *F*(3,72) = 62.22, *p <* 0.001), post hoc analyses showed that the level of anger and disappointment were higher for CD outcome than the other three outcomes (anger: *ps <* 0.001; disappointment: *ps* < 0.001) and higher in the gain than the loss context (*p* = 0.023). For ratings of shame and guilt, there were significant main effects of outcome (shame: *F*(3,72) = 62.88, *p <* 0.001; guilt: *F*(3,72) = 76.78, *p <* 0.001), post hoc analyses showed that the level of shame and guilt were higher for DC outcome than the other three outcomes (shame: *ps <* 0.001; guilt: *ps* < 0.001).

### fMRI results

#### ROI analyses

**Fig 3:**
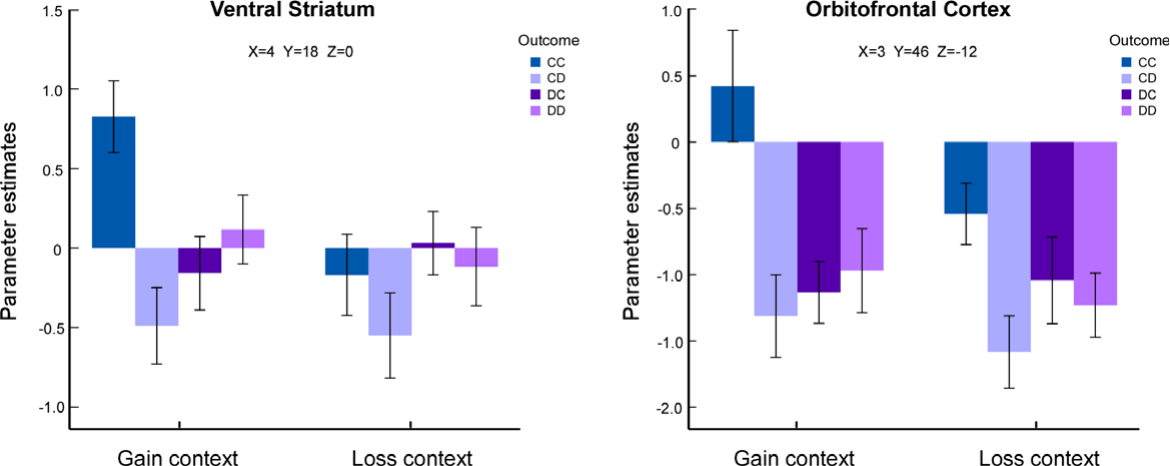
ROI analysis of the parameter estimates of the ventral striatum and orbitofrontal cortex for eight experimental conditions.

Parameter estimates across the ventral striatum (MNI 4, 18, 0) and the orbitofrontal cortex (MNI 3, 46, -12) reported in Rilling et al. [6, 7] were extracted. For the ventral striatum, a 2 (context: loss vs. gain) × 4 (outcome: CC, CD, DC, and DD) repeated measures ANOVA revealed significant main effects of context (*F*(1,17) = 4.57, *p =* 0.047) and outcome (*F*(3,51) = 4.85, *p =* 0.005), indicating greater activities in the gain than in the loss context and increased activities for CC outcome than the other three. A significant interaction was also found (*F*(3,51) = 4.61, *p =* 0.006). We further examined the effect of outcome within each context. Post hoc analysis showed that activities is greater for CC outcome than the other three in the gain context (*ps < 0*.*01*), whereas there was no significant difference between outcomes in the loss context (Fig 3).

For the orbitofrontal cortex, similar ANOVA was carried out. The result showed the main effect of outcome (*F*(3,51) = 6.40, *p =* 0.001), Post hoc analyses indicated greater activities for CC outcome than the other three (*ps <* 0.05). There was no significant main effect of context or context-by-outcome interaction (Fig 3).

#### Whole-brain analyses

Compared with the other three outcomes, experiencing CC outcome activated left ventral striatum (MNI -6–8 22), left orbital frontal cortex (MNI -6 54–2), left anterior cingulate cortex (MNI -4 40–6), left dorsal medial prefrontal cortex (MNI -12 58 16), left lingual gyrus (MNI -18–86–10), right calcarine gyrus (MNI 16–92 4), and bilateral superior temporal gyrus (MNI -40–38 16 and 70–26 6). The reverse contrast showed greater activities in right lingual gyrus (MNI 10–78 0) and left inferior parietal lobule (MNI -26–54 52) (Table 1).

**Table 1:** Regions Showing Main Effects of Outcome.

		Coordinates				
Brain Region	Side	x	y	z	*t*-value	Voxels
***CC—A***						
Dorsal Medial Prefrontal Cortex	L	-20	42	38	8.44	6265
* Orbital Frontal Cortex*	*L*	*-6*	*54*	*-2*	*7*.*07*	
* Anterior Cingulate Cortex*	*L*	*-4*	*40*	*-6*	*6*.*35*	
Lingual Gyrus	L	-18	-86	-10	5.86	725
Calcarine Gyrus	R	16	-92	4	5.64	486
Superior Temporal Gyrus	L	-40	-38	16	5.44	1275
Superior Temporal Gyrus	R	70	-26	6	4.75	1014
Ventral Striatum	L	-6	-8	22	4.04	188
***A—CC***						
Lingual Gyrus	R	10	-78	0	6.36	837
Inferior Parietal Lobule	L	-26	-54	52	4.38	271

*Note*. Coordinates (mm) are in MNI space. L = left hemisphere; R = right hemisphere. All reported clusters are cluster-level family wise error (FWE) corrected for multiple comparisons at *p* < 0.05 with an underlying voxel-level of *p* < 0.001 (uncorrected).

Data analyses revealed that dorsal medial prefrontal cortex (MNI -12 58 16) and cuneus (MNI 16–80 28) survived by contrasting gain trials with loss trials, whereas no region was activated in the reverse contrast (Table 2).

**Table 2:** Regions Showing Main Effects of Context.

		Coordinates				
Brain Region		x	y	z	*t*-value	Voxels
***Gain—Loss***						
Dorsal Medial Prefrontal Cortex	L	-12	58	16	5.69	1187
Cuneus	R	16	-80	28	4.89	327
Ventral Striatum	R	14	18	2	4.52	182
***Loss—Gain***						
No regions						

*Note*. Coordinates (mm) are in MNI space. L = left hemisphere; R = right hemisphere. All reported clusters are cluster-level family wise error (FWE) corrected for multiple comparisons at *p* < 0.05 with an underlying voxel-level of *p* < 0.001 (uncorrected).

**Fig 4:**
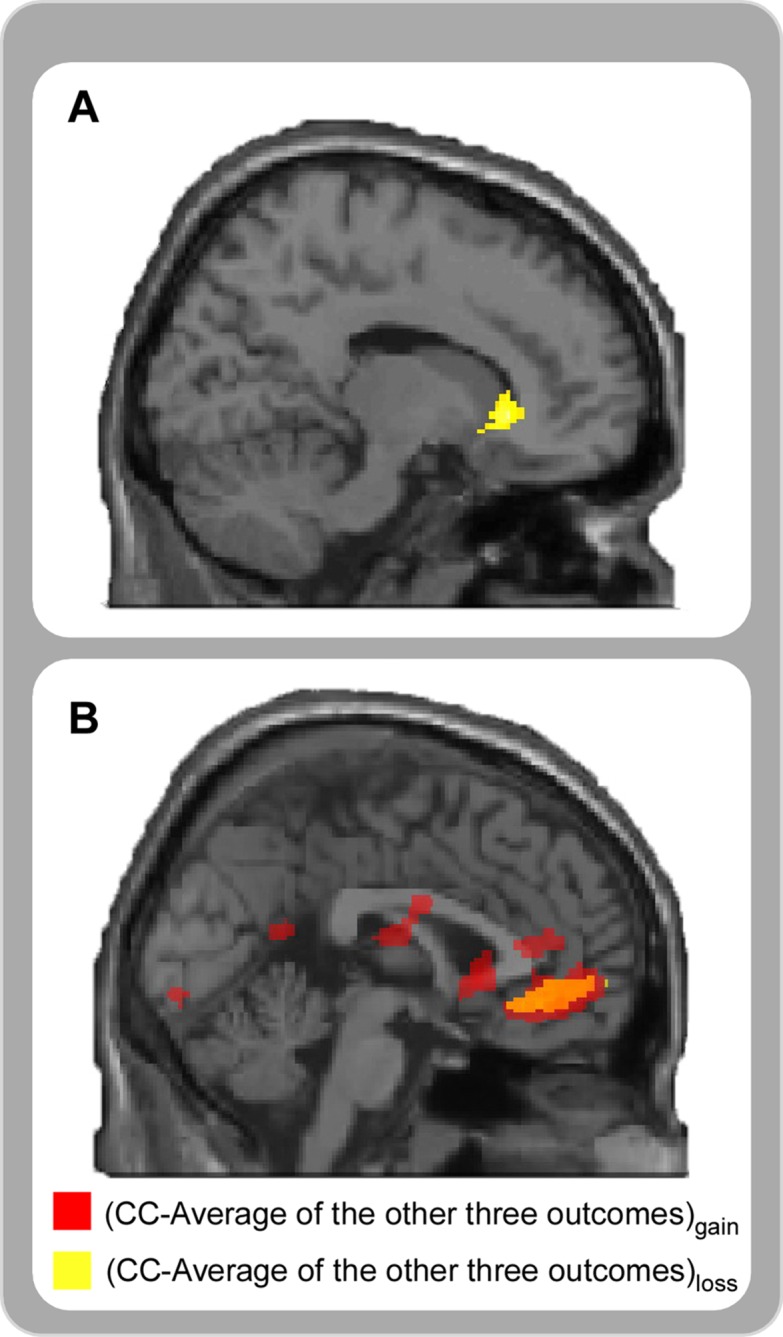
(A) Brain activities in Outcome × Context interaction (*p* < 0.001, uncorrected). (B) Clusters in OFC overlapped between the simple effect of outcome in gain context and in loss context (*p* < 0.001, uncorrected).

Interaction between context and cooperation was computed by the (CC—Average of the other three outcomes)_gain_—(CC—Average of the other three outcomes)_loss_. Results showed that bilateral ventral striatum (MNI -4 18 0 and 14 26–2) and inferior temporal gyrus (MNI -38–28–14) were activated (Table 3; Fig 4A), whereas no region was activated in the reverse contrast.

**Table 3:** Regions Showing Outcome × Context Interactions.

		Coordinates				
Brain Region	Side	x	y	z	*t*-value	Voxels
***(CC-A)***_*Gain*_*—****(CC-A)***_*Loss*_						
Inferior Temporal Gyrus	L	-38	-28	-14	5.22	633
Ventral Striatum	R	14	26	-2	4.36	348
* *Ventral Striatum	*L*	*-4*	*18*	*0*	*4*.*19*	
***(CC-A)***_*Loss*_*—****(CC-A)***_*Gain*_						
No regions						

*Note*. Coordinates (mm) are in MNI space. L = left hemisphere; R = right hemisphere. All reported clusters are cluster-level family wise error (FWE) corrected for multiple comparisons at *p* < 0.05 with an underlying voxel-level of *p* < 0.001 (uncorrected).

To further clarify whether there was a common neural mechanisms, we overlapped the simple effect of outcome in gain context (CC—Average of the other three outcomes)_gain_ with the simple effect of outcome in loss context (CC—Average of the other three outcomes)_loss_. The cluster located in the orbital frontal cortex overlapped between these two contrasts (Fig 4B).

## Discussion

We employed a modified version of the one-shot PD game to investigate how loss and gain context modulated brain responses to cooperation. Consistent with previous studies[4, 5, 14, 15], even in this anonymous and one-shot PD game, participants still choose to cooperate in nearly half of the experiment trials, and they cooperated more in the gain than in the loss context. At the neural level, both the ROI and whole brain analysis revealed more activities in the ventral striatum and the orbitofrontal cortex for mutual cooperation relative to the other three outcomes. When focusing on modulation of cooperation response by context, we found that the ventral striatum only showed cooperation-specific activations in the gain context, but not in the loss context. And overlap analysis showed that cooperation-specific activations in the orbitofrontal cortex were not affected by context.

Postscan emotion rating showed that mutual cooperation was the most personally satisfying outcome. Specifically, participants experienced high level positive emotions such as happiness and trust for CC outcome, while the most profitable DC outcome provoked negative feelings of shame and guilt. Previous studies using PD game had also revealed that the ventral striatum and orbitofrontal cortex were activated more by mutual cooperation than by a same monetary reward in a nonsocial context[6, 14]. Combined with the neuroimaging evidence linking the ventral striatum and orbitofrontal cortex to processing the subjective value of fairness and cooperation[6, 8, 9, 16, 17], our data suggest that increased activation levels in the ventral striatum and orbitofrontal cortex with the CC outcome may relate to the rewarding experience from a mutually cooperative social interaction. In addition, our results are also consistent with previous findings that reward-related neural activity was greater for positive reward prediction errors [18–21], or a discrepancy that an actual reward is superior to what is predicted. In the one-shot PD game, participants could never know for certain whether partner would cooperate or not, so the game outcomes were always of an unpredictable nature. Therefore, when participants chose to cooperate, compared with CD outcome, CC outcome involves a positive reward prediction error [6, 14].

We further studied the impact of context on participants' behavioral and neural responses to cooperation. It was revealed that participants cooperated more and experienced higher level of happiness for cooperation in the gain context than in the loss context. Parallel with this, cooperation-specific activations in the ventral striatum only existed in the gain context. Our findings were consisted with the evidence from a recent functional neuroimaging study [22], in which the authors employed a modified version of the Ultimatum Game to investigate how brain differentially responds to fairness in loss and gain contexts. They found the association between activation in ventral striatum and fairness was weakened in the loss context. Except of being involved in processing the subjective value of fairness and cooperation [6, 16, 17], the ventral striatum is also known for its vital contribution to the cerebral representation of the primary reinforcement and monetary reward [23–25]. Therefore, under the shadow of monetary loss, the activation of ventral striatum might be weakened.

However, the orbitofrontal cortex was also activated more for mutual cooperation relative to the other three outcomes, and this cooperation-specific activation was not affected by context. Previous studies have identified the significant role of the orbitofrontal cortex in mediating the mental pleasure related to the secondary reinforcement with social implications [26, 27]. For example, a recent study[28] revealed that processing moral beauty is related to activations in the cortical reward region of the orbitofrontal cortex, whereas facial beauty involved both cortical and subcortical reward regions (the orbitofrontal cortex and the putamen). Based on previous and our findings, we speculated that, compared with the ventral striatum, the orbitofrontal cortex might be more sensitive to social reward involving moral implication. Taken together, the activation of the orbitofrontal cortex observed in our experiment might be involved in processing purer social reward of cooperation which was immune to the impact of loss context.

Dorsal medial prefrontal cortex and rostral anterior cingulate cortex activities were also found when identifying activations associated with mutual cooperation. Previous studies have revealed that the DMPFC was involved in the evaluation of self-referential stimuli and moral judgment [29–32]. The rACC with dense projections to orbitofrontal cortex and striatum is part of a wider network that mediates reinforcement and reward preference [33–35]. Thus, it is plausible that rACC activities in the present study might be related to the reinforcement of cooperation and DMPFC activities might be associated with moral evaluation of self.

In conclusion, the current study further illustrated that the context of loss and gain modulate people’s behavioral and neural responses to social cooperation. Results showed that, at the behavioral level, the mutual cooperation was the most satisfying outcome for participants but they cooperated more in the gain context than the loss context. At the neural level, cooperation-specific activations in the ventral striatum only existed in the gain context, but activations in the orbitofrontal cortex were not affected by context. Together, our data indicated that, even in the one-shot interaction under loss context, participants still derive higher hedonic value from the mutual cooperation, but the loss context weakened the association between the ventral striatum activation and mutual cooperation.

Future studies might further examine the context effect on neural responses to social cooperation from two aspects. Firstly, given some previous studies reported men and women behaved differently in the PD game, future researches might explore gender differences in neural responses to social cooperation in both contexts. Secondly, following conventional approach, participants were initially endowed with a sum of money as basic payment in the current study. Thus, the loss context in the current study was defined by a relative loss frame. Future studies might evaluate the context effect on neural responses to social cooperation with absolute loss context.

## Supporting Information

S1 File Cooperation rates in the gain and loss context. (XLSX)

S2 File Emotion ratings for each outcome of the PD game in the gain and loss context. (XLSX)
